# Supplementary material for: Involvement of an IgE/Mast cell/B cell amplification loop in abdominal aortic aneurysm progression
Source: PLoS One. 2023 Dec 6;18(12):e0295408. doi: 10.1371/journal.pone.0295408 (PMC10699626; doi:10.1371/journal.pone.0295408)
Supplement: S1 Table — (PDF) [file pone.0295408.s001.pdf]

Table S1: Patients'characteristics.

	All (n=57)	Histology (n=25)	FC (n=25)	CM (n=37)
**Age (yrs)**	68 +/- 2	70 +/- 2	73 +/- 2	68 +/- 2
**Male**	86%	92%	92%	84%
**Aneurysm location**				
Suprarenal	2.5%	5%	0%	4%
Juxtarenal	2.5%	5%	0%	0%
Subrenal	59%	58%	83%	57%
Subrenal+iliac	36%	32%	17%	39%
**Maximum aortic diameter (cm)**	61 +/- 2	61 +/- 3	67 +/- 4	63 +/- 3
**Clinical Features**				
Diabetes	5%	5%	0%	7%
Hypertension	81%	89%	86%	87%
Hyperlipidaemia	40%	47%	58%	37%
Smoking	83%	84%	86%	80%
**Anti-coagulants**	15%	17%	27%	15%

Values are mean +/- SEM or %, for all AAA samples, or samples according to processing (some samples were used for several kind of analysis). FC: flow cytometry analysis (MCs and/or B cells); CM: conditioned medium (IgE concentration and/or MC stimulation).
