# Supplementary material for: Involvement of an IgE/Mast cell/B cell amplification loop in abdominal aortic aneurysm progression
Source: PLoS One. 2023 Dec 6;18(12):e0295408. doi: 10.1371/journal.pone.0295408 (PMC10699626; doi:10.1371/journal.pone.0295408)
Supplement: S2 Table — (PDF) [file pone.0295408.s002.pdf]

Table S2: NAA healthy donors'characteristics.

	All (n=39)	Histology (n=7)	FC (n=16)	CM (n=24)
**Age (yrs)**	55 +/- 3	53 +/- 9	55 +/- 5	56 +/- 4
**Male**	61%	80%	56%	61%
**Atherosclerotic lesion**				
None	28%	29%	31%	25%
Fatty streak	44%	43%	31%	54%
Fibrolipidic	26%	29%	31%	17%
Intraplaque hemorrhage	3%	0%	6%	4%

Values are mean +/- SEM or %, for all NAA samples, or samples according to processing (some samples were used for several kind of analysis). FC: flow cytometry analysis (MCs and/or B cells); CM: conditioned medium (IgE concentration and/or mast cell stimulation).
