# Supplementary material for: Involvement of an IgE/Mast cell/B cell amplification loop in abdominal aortic aneurysm progression
Source: PLoS One. 2023 Dec 6;18(12):e0295408. doi: 10.1371/journal.pone.0295408 (PMC10699626; doi:10.1371/journal.pone.0295408)
Supplement: S3 Table — (PDF) [file pone.0295408.s003.pdf]

Table S3: ApoE-RMB survival and aneurysm occurrence.

	PBS (n = 17)	DT (n = 18)	p-value
**Aneurysm**	n = 7 (47%)	n = 8 (44%)	0.85
**Death***	n = 0 (0%†)	n = 2 (25%†)	0.15

*, dead mice presented aneurysms and died 21 days after the beginning of AngII infusion (7 days after DT treatment). †, % of mice with AAA.
