# Supplementary material for: Involvement of an IgE/Mast cell/B cell amplification loop in abdominal aortic aneurysm progression
Source: PLoS One. 2023 Dec 6;18(12):e0295408. doi: 10.1371/journal.pone.0295408 (PMC10699626; doi:10.1371/journal.pone.0295408)
Supplement: S1 Fig — (PDF) [file pone.0295408.s004.pdf]

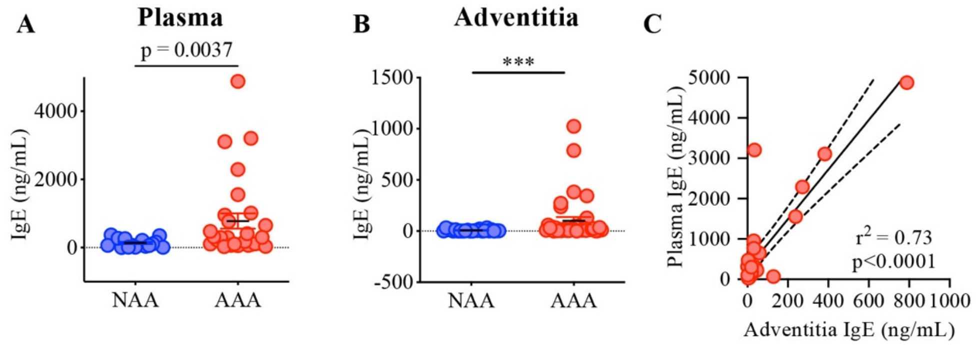

Fig S1

Fig S1. IgEs are elevated in the plasma and adventitia of AAA patients.

IgEs were titrated in the plasma (A) and conditioned medium from adventitia (B) of NAA organ donors and AAA patients. Mann-Whitney tests were used to compare groups (***: $p < 0.001$). Correlation between plasmatic and adventitial IgE concentration in AAA patients (C). r^2 and p-values from Pearson correlation analysis are indicated.
