# Supplementary material for: Involvement of an IgE/Mast cell/B cell amplification loop in abdominal aortic aneurysm progression
Source: PLoS One. 2023 Dec 6;18(12):e0295408. doi: 10.1371/journal.pone.0295408 (PMC10699626; doi:10.1371/journal.pone.0295408)
Supplement: S2 Fig — (PDF) [file pone.0295408.s005.pdf]

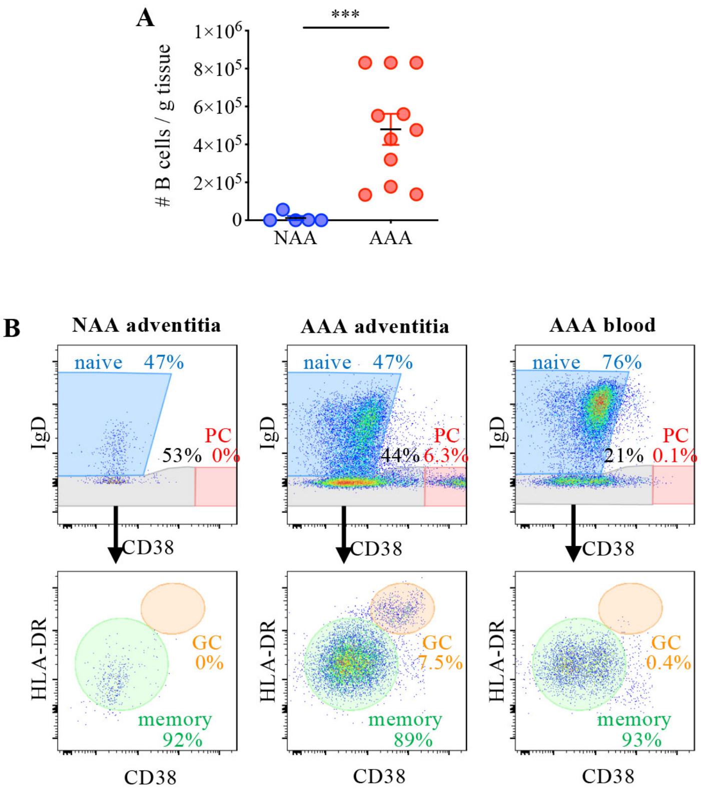

Fig S2

Fig S2. GC B cells and plasma cells are elevated in AAA adventitia.

Adventitia from NAA organ donors and AAA patients were digested and analysed by flow cytometry after the addition of fluorescent count beads. (A) B cells were identified as singlet, autofluorescent$^-$, live CD45$^+$ CD19$^+$ cells, and their number was calculated in each sample, showing a statistically significant increase of B cells in AAA samples. ***, $p < 0.001$, Mann-Whitney test. (B) B cells were identified as in (A), and subsets were defined as follows: naïve B cells, IgD$^+$ CD38$^-$; plasma cells (PC), IgD$^-$ CD38^{hi}; germinal centre B cells (GC), IgD$^-$ CD38$^+$ HLA-DR^{hi}; memory B cells, IgD$^-$ CD38$^-$ HLA-DR$^+$. Representative samples show that GC B cells and plasma cells were present in the adventitia of AAA, while they were barely detected in the matched blood of the AAA patient, or in the adventitia of NAA.
