# Supplementary material for: Involvement of an IgE/Mast cell/B cell amplification loop in abdominal aortic aneurysm progression
Source: PLoS One. 2023 Dec 6;18(12):e0295408. doi: 10.1371/journal.pone.0295408 (PMC10699626; doi:10.1371/journal.pone.0295408)
Supplement: S3 Fig — (PDF) [file pone.0295408.s006.pdf]

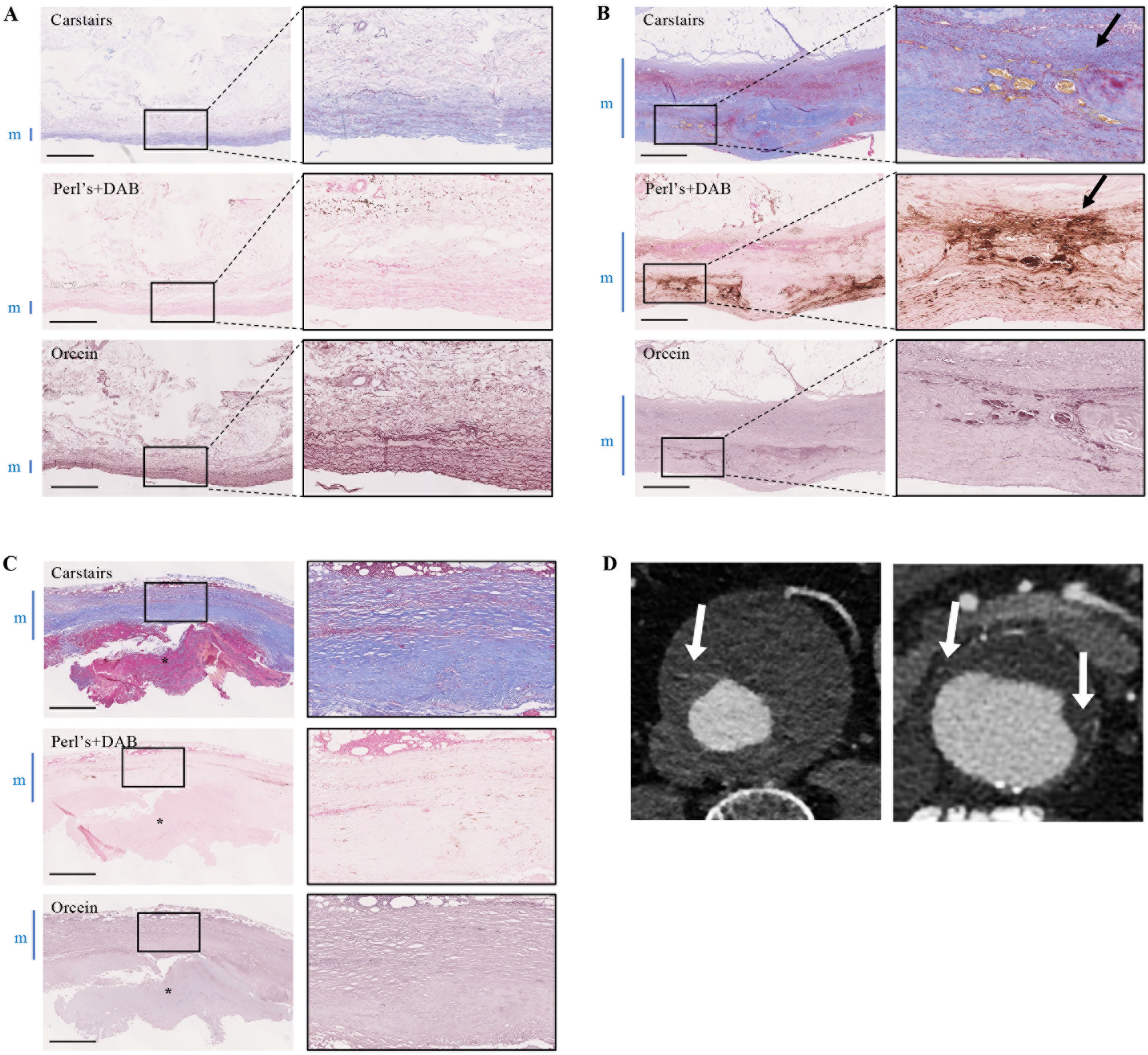

Fig S3

Fig S3. AAAs with ancient or no intramural haematomas.

Histological staining of NAAs (A) and AAAs (B-C). Serial sections were stained with Carstairs's stain, Perl's+DAB stain and Orcein stain as in Fig 1C. The squares frame the localisation of the details shown in the insets. The lumen is at the bottom of each picture, and the media (m) is indicated by the blue bar on the side of the pictures. In A, intact elastin fibers can be seen in the media on the Orcein stain. In B, black arrows indicate iron deposits in the

absence of red blood cells, suggestive of an ancient intramural haematoma. An intraluminal thrombus (*) can be seen in C, where no signs of intramural haematoma were seen. Scale bar: 1 mm. (D) Contrast-enhanced tomography angiograms of AAAs. White arrows: blood disruption from the aortic lumen to the aortic wall through the intraluminal thrombus.
