# Supplementary material for: Involvement of an IgE/Mast cell/B cell amplification loop in abdominal aortic aneurysm progression
Source: PLoS One. 2023 Dec 6;18(12):e0295408. doi: 10.1371/journal.pone.0295408 (PMC10699626; doi:10.1371/journal.pone.0295408)
Supplement: S4 Fig — (PDF) [file pone.0295408.s007.pdf]

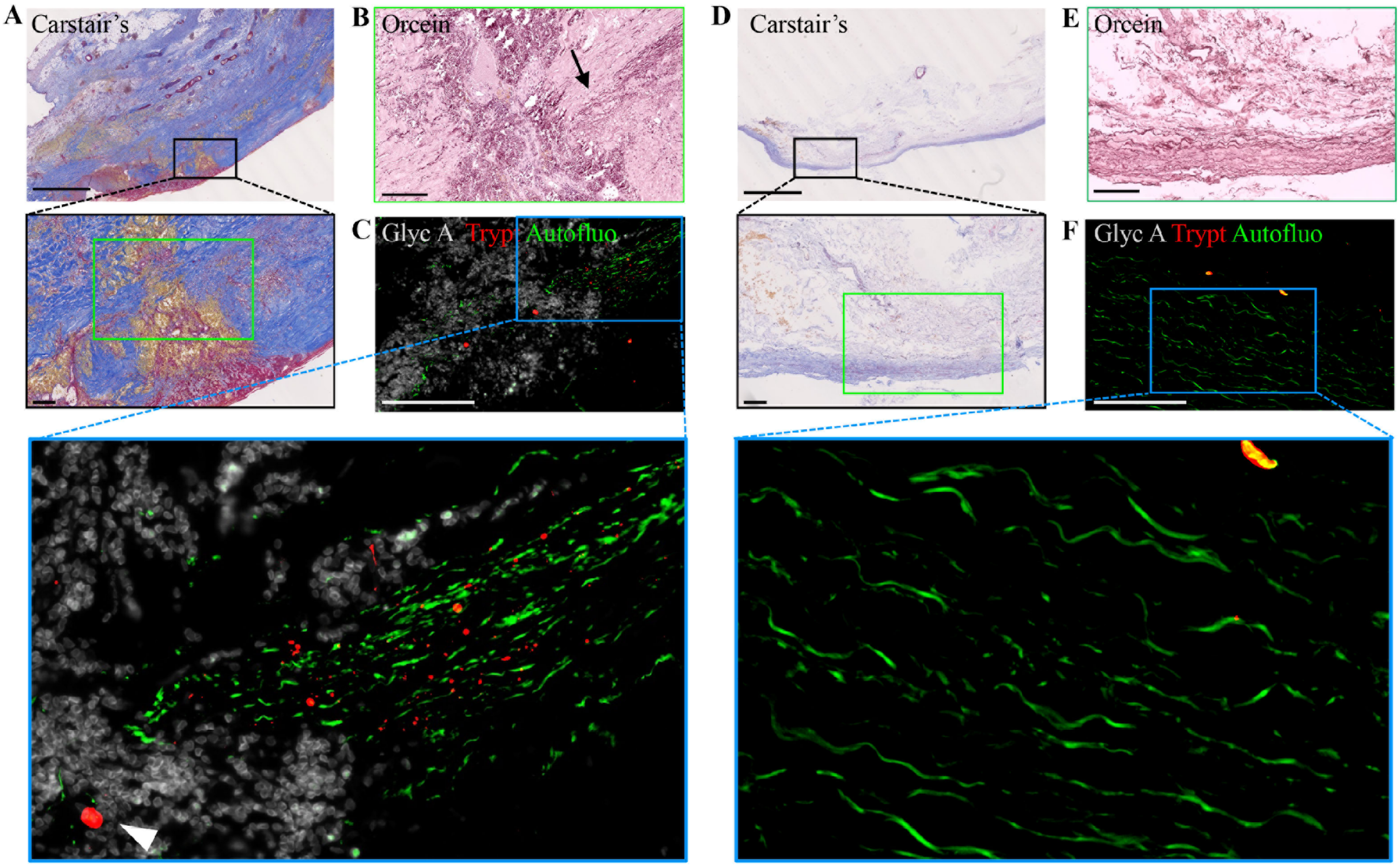

Fig S4

Fig S4. Proximity of micro-fissures and MC degranulation in human AAAs.

Serial sections of an AAA sample with a micro-fissure (A-C) and from a NAA sample (D-F) were stained with: Carstair's stain (A and D); orcein to reveal elastin fibres (B and E); and tryptase (red) and glycophorin A (gray) to detect MCs/tryptase$^+$ granules and red blood cells, respectively (C and F; the elastin fibres were autofluorescent: green). The pictures in B and C, and E and F, correspond to the green insets in A and D, respectively. The entry site of blood into the aortic wall in the AAA sample can be observed on the Carstair's stain (A, red blood cells appear in yellow). The blood entry site visible on the Carstair's stain (A) and with the glycophorin A stain (C) was associated with degraded elastin fibres (black arrow on orcein stain in B) and the presence of MCs (white arrowhead) and tryptase$^+$ granules (C). Note the normal aspect of the elastin fibers (E) as well as the absence of tryptase and red blood cells (F) in the control. Scale bars: A and D 2.5 mm; others: 200 µm.
