# Supplementary material for: Involvement of an IgE/Mast cell/B cell amplification loop in abdominal aortic aneurysm progression
Source: PLoS One. 2023 Dec 6;18(12):e0295408. doi: 10.1371/journal.pone.0295408 (PMC10699626; doi:10.1371/journal.pone.0295408)
Supplement: S5 Fig — (PDF) [file pone.0295408.s008.pdf]

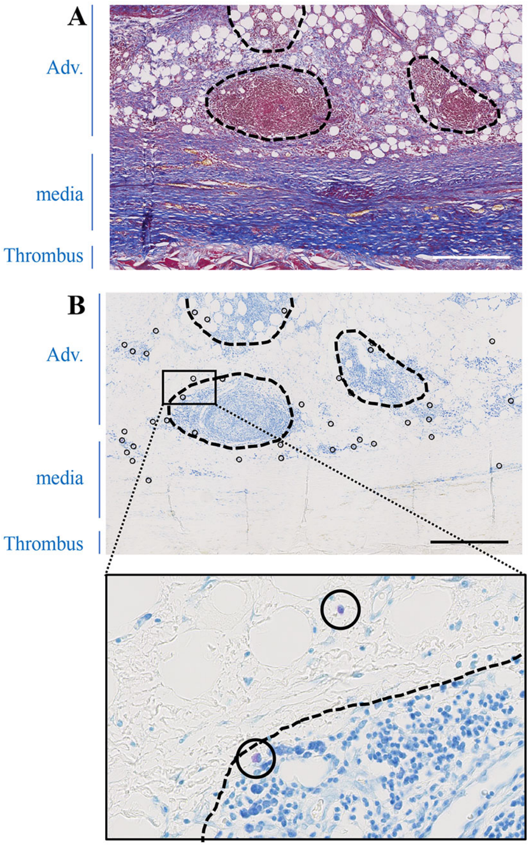

Fig S5

Fig S5. MCs accumulate in the adventitia of human AAAs.

Carstairs' stain (A) and toluidine blue stain (B-C) on serial sections of a representative micro-fissured AAA sample. MCs appear purple on toluidine blue stain (indicated with black circles). TLOs are circled with a dotted line. Scale bar: 500 µm.
