# Supplementary material for: Involvement of an IgE/Mast cell/B cell amplification loop in abdominal aortic aneurysm progression
Source: PLoS One. 2023 Dec 6;18(12):e0295408. doi: 10.1371/journal.pone.0295408 (PMC10699626; doi:10.1371/journal.pone.0295408)
Supplement: S6 Fig — (PDF) [file pone.0295408.s009.pdf]

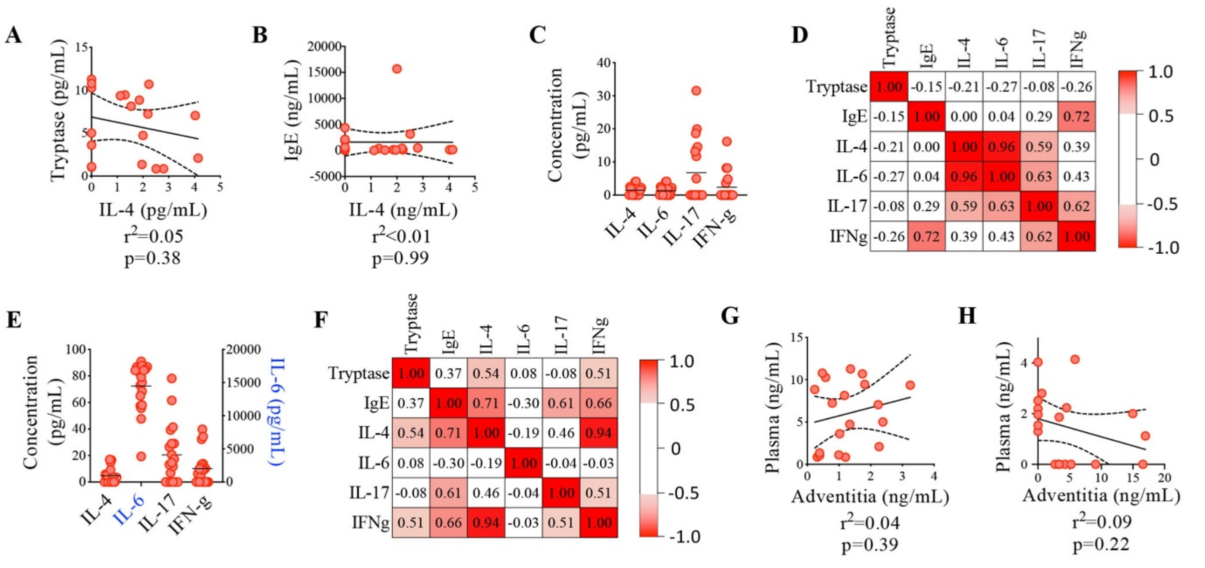

Fig S6

Fig S6. No correlation between tryptase, IgE and IL-4 in the plasma of AAA patients.

IL-4, tryptase, and IgE concentrations were measured in the plasma (A-D, G-H)) and adventitia (E-H) of AAA patients (same patients as in Fig 3E-F). Correlations between plasma tryptase, IgEs and IL-4 (A, B). Plasma concentration of cytokines (C). r-values from Pearson correlation analysis between plasma molecules (D). Adventitia concentration of cytokines (E). r-values from Pearson correlation analysis between adventitia molecules (F). Correlation between plasma and adventitia tryptase (G) and IL-4 (H). r^2 and p-values from Pearson correlation analysis are indicated (A, B, G, H). TNF-alpha and IL-10 were below the range of detection.
