# Supplementary material for: Involvement of an IgE/Mast cell/B cell amplification loop in abdominal aortic aneurysm progression
Source: PLoS One. 2023 Dec 6;18(12):e0295408. doi: 10.1371/journal.pone.0295408 (PMC10699626; doi:10.1371/journal.pone.0295408)
Supplement: S7 Fig — (PDF) [file pone.0295408.s010.pdf]

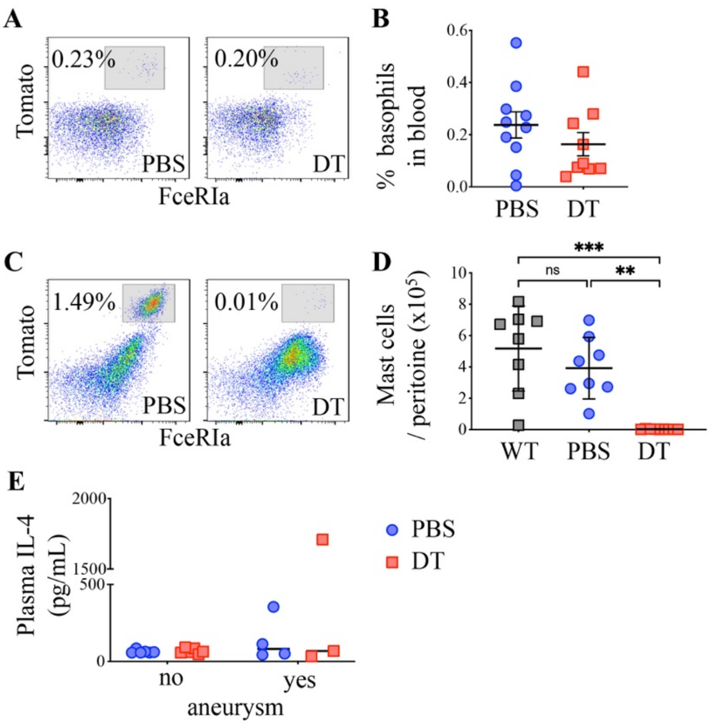

Fig S7

Fig S7. Repopulation of basophils in the blood and MCs in the peritoneum after DT depletion in ApoE RMB mice.

ApoE-RMB or ApoE mice were treated as in Fig 4A. At day 14 after DT (n=9) or PBS (n=10) injection (day 28 of Ang II infusion), mice were sacrificed. (A) Blood basophils were identified among singlet cells, as CD45$^+$ Live/Dead$^-$ CD3$^-$ CD19$^-$ FcεRIa$^+$ Tomato$^+$ cells (22). (B) Percentage of basophils within singlet cells, CD45$^+$ Live/Dead$^-$ cells. ***: p<0.001, Mann-Whitney test. (C) Peritoneal MCs were identified among singlet cells as CD45$^+$ Live/Dead$^-$ CD3$^-$ CD19$^-$ FcεRIa$^+$ CD117$^+$ (Tomato$^+$) cells (22). (D) Number of MCs (CD45$^+$ Live/Dead$^-$ CD3$^-$ CD19$^-$ FcεRIa$^+$ CD117$^+$) per peritoneal lavage. WT: DT-treated ApoE-deficient mice. ***: p<0.001, **: p<0.01, Kruskal Wallis test followed by Dunn's multiple comparison. Data are representative of two experiments. (E) Plasmatic concentration of IL-4, depending of presence of aneurysm and treatment. Analysis of covariance did not show a statistical effect of

presence of aneurysm or treatment. TNF-alpha, IL-6 IFN-gamma and IL-1beta were not detectable.
