# Supplementary material for: Involvement of an IgE/Mast cell/B cell amplification loop in abdominal aortic aneurysm progression
Source: PLoS One. 2023 Dec 6;18(12):e0295408. doi: 10.1371/journal.pone.0295408 (PMC10699626; doi:10.1371/journal.pone.0295408)
Supplement: S8 Fig — (PDF) [file pone.0295408.s011.pdf]

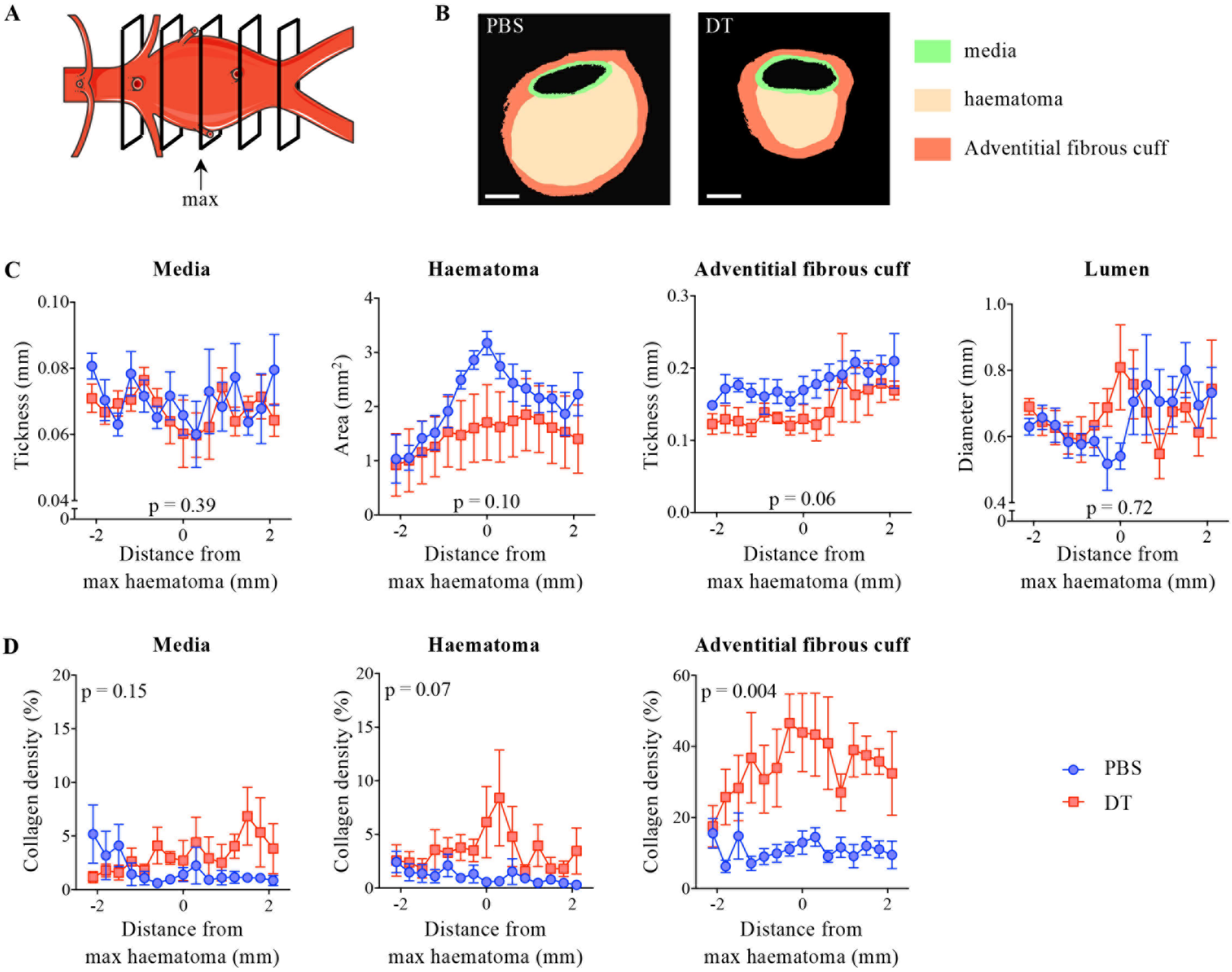

Fig S8

Fig S8. MCs promote aneurysm expansion after dissection in ApoE-RMB mice.

(A) Sections taken at different levels (every 300 µm) from each aneurysm were used for analysis. (B) Vascular wall layers were defined by computer-assisted morphometry on Sirius red-stained cross-sections (Fig 4E) of aneurysm (scale bar: 500 µm). Size (C) and collagen density (D) of the different layers were calculated by computer-assisted morphometry, and plotted relatively to the distance from the layer with the largest haematoma. Mean +/- standard error; p-values for temperature effect in mixed-model (REML) analysis.
