# Supplementary figures and images for: Involvement of an IgE/Mast cell/B cell amplification loop in abdominal aortic aneurysm progression
Source: PLoS One. 2023 Dec 6;18(12):e0295408. doi: 10.1371/journal.pone.0295408 (PMC10699626; doi:10.1371/journal.pone.0295408)

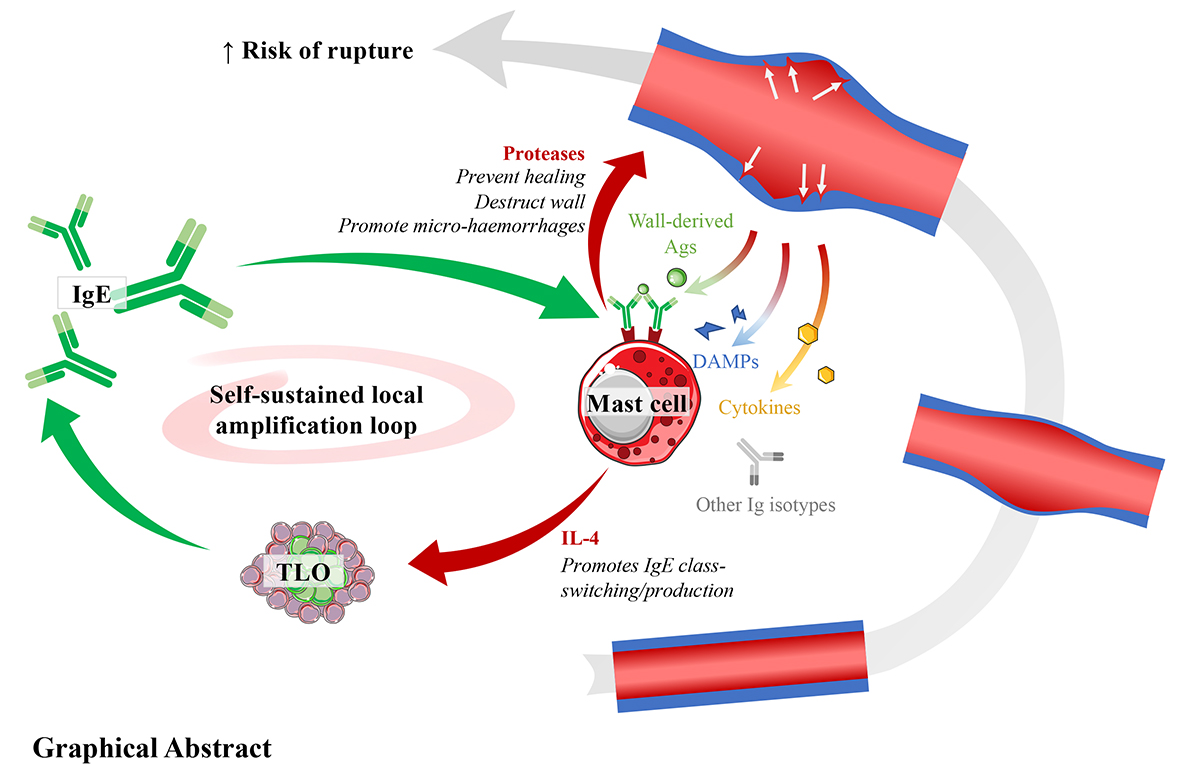

Supplement: S1 Graphical abstract — (TIF) [file pone.0295408.s013.tif]
